# Preparing and Mounting Sections of Brain

**Published:** 1889-12

**Authors:** 


### Preparing and Mounting Sections of Brain.

The method which we have found to give the best results in preparing, staining, and mounting sections of brain and spinal cord is that of Sigismund Freund, of the General Hospital, at Vienna. We gave it in the *National Druggist* several years ago, but repeat it now in answer to a request from a correspondent. It is as follows: "The material is to be hardened first in a solution of bichromate of potash, or in Erlich's fluid (bichromate of potash, 5 parts; sulphate of copper, 1 part; distilled water, 200 parts), and afterward in alcohol. The sections are floated in distilled water, and washed thoroughly. A staining fluid is made by mixing equal parts of a 1 per cent. solution of chloride of gold in distilled water and strong alcohol. The washed ssctions are put in this and allowed to remain from three to five hours. From this stain they are removed to a fixing solution consisting of caustic potash, 1 part; distilled water, 5 or 6 parts, and allowed to remain in it for two or three minutes. From this solution they are removed directly to a 10 per cent. solution of iodide of potash, where they immediately assume a rose color, which, in the course of a few minutes, deepens into red. At this point, if the sections are from adult tissues, the preparatory stage ceases, and they may be transferred to alcohol and mounted in the usual way, but the brain and spinal cord of an embryo are far too delicate to be handled in this way, and the author treats them as follows: The preparations are brought upon a glass slide, spread by means of a camel's hair brush, and gently dried by covering (without pressure) with a piece of filtering paper. If the sections be very thin and soft, even this must be avoided, for the fibres of the paper would leave traces on the surface of the sections, and render them unfit for the study of the nervous elements. Nothing else can be done but to apply a piece of filter paper to the edges of the stained preparation lying on the slide, and to draw off the alkaline fluid in this way. This is by far the most tedious stage of the process, yet it is always possible to avoid shrinking, and to preserve the most sensitive preparations. The sections, nearly dried, are allowed to remain in water a few minutes and then transferred to alcohol, and mounted in the usual manner in Canada balsam.—*National Druggist*, July 1, '89.

---

OVERCROWDING OF THE PROFESSIONS IN GERMANY.—Prof. Lexis, of Berlin, has been writing a pamphlet on the undue increase in all the learned professions. He says there are twice as many students as can hope to make a living by the professions which they are preparing to enter.

